# Impact of e-cigarette retail displays on attitudes to smoking and vaping in children: an online experimental study

**DOI:** 10.1136/tobaccocontrol-2021-056980

**Published:** 2022-04-13

**Authors:** Anna K M Blackwell, Mark A Pilling, Katie De-Loyde, Richard W Morris, Laura A Brocklebank, Theresa M Marteau, Marcus R Munafò

**Affiliations:** 1 School of Psychological Science, University of Bristol, Bristol, UK; 2 Behaviour and Health Research Unit, University of Cambridge, Cambridge, UK; 3 Bristol Medical School: Population Health Sciences, University of Bristol, Bristol, UK; 4 Department of Behavioural Science and Health, University College London, London, UK

**Keywords:** electronic nicotine delivery devices, environment, public policy

## Abstract

**Objectives:**

To estimate the impact of electronic cigarette (e-cigarette) retail display exposure on attitudes to smoking and vaping (susceptibility to tobacco smoking and using e-cigarettes, and perceptions of the harms of smoking and e-cigarette use).

**Design:**

Between-subjects randomised experiment using a 2 (e-cigarette retail display visibility: high vs low)×2 (proportion of e-cigarette images: 75% vs 25%) factorial design.

**Setting:**

Online via the Qualtrics survey platform.

**Participants:**

UK children aged 13–17 years (n=1034), recruited through a research agency.

**Intervention:**

Participants viewed 12 images of retail displays that contained e-cigarette display images or unrelated product images. E-cigarette display images were either high or low visibility, based on a conspicuousness score. Participants were randomised to one of four groups, with e-cigarette display visibility and proportion of e-cigarette images, compared with images of unrelated products, manipulated: (1) 75% e-cigarettes, high visibility; (2) 25% e-cigarettes, high visibility; (3) 75% e-cigarettes, low visibility; (4) 25% e-cigarettes, low visibility.

**Main outcome measures:**

The primary outcome was susceptibility to smoking (among never smokers only). Secondary outcomes were susceptibility to using e-cigarettes (among never vapers only), and perceptions of smoking and e-cigarette harm (all participants).

**Results:**

Neither e-cigarette retail display visibility, nor the proportion of e-cigarette images displayed, appeared to influence susceptibility to smoking (visibility: OR=0.84, 95% CI 0.62 to 1.13, p=0.24; proportion: OR=1.34, 95% CI 1.00 to 1.82, p=0.054 (reference: low visibility, not susceptible)).

Planned subgroup analyses indicated that exposure to a higher proportion of e-cigarette images increased susceptibility to smoking among children who visited retail stores more regularly (n=524, OR=1.59, 95% CI 1.04 to 2.43, p=0.034), and those who passed the attention check (n=880, OR=1.43, 95% CI 1.03 to 1.98, p=0.031).

In addition, neither e-cigarette retail display visibility nor the proportion of e-cigarette images displayed, appeared to influence susceptibility to using e-cigarettes (visibility: OR=1.07, 95% CI 0.80 to 1.43, p=0.65; proportion: OR=1.22, 95% CI 0.91 to 1.64, p=0.18).

Greater visibility of e-cigarette retail displays reduced perceived harm of smoking (mean difference (MD)=−0.19, 95% CI −0.34 to −0.04, p=0.016). There was no evidence that the proportion of e-cigarette images displayed had an effect (MD=−0.07, 95% CI −0.22 to 0.09, p=0.40).

Perceived harm of e-cigarette use did not appear to be affected by e-cigarette retail display visibility (MD=−0.12, 95% CI −0.28 to 0.05, p=0.16) or by the proportion of e-cigarette images displayed (MD=−0.10, 95% CI −0.26 to 0.07, p=0.24).

**Conclusions:**

There is no evidence in the full sample to suggest that children’s susceptibility to smoking is increased by exposure to higher visibility e-cigarette retail displays, or to a higher proportion of e-cigarette images. However, for regular store visitors or those paying more attention, viewing a higher proportion of e-cigarette images increased susceptibility to smoking. In addition, viewing higher visibility e-cigarette images reduced perceived harm of smoking. A review of the current regulatory discrepancy between tobacco and e-cigarette point-of-sale marketing is warranted.

**Trial registration number:**

ISRCTN18215632.

## Introduction

Many countries—including the UK, Iceland, Canada and Australia—have banned tobacco retail displays, given evidence linking them to increased purchasing of, and reduced abstinence from, smoking among adults, and increased smoking susceptibility and initiation among young people.^1–3^ Evaluations of these bans suggest they can facilitate a change in perceptions of smoking and reduce impulse purchasing,^1–3^ as well as reduce smoking susceptibility among young people.^4^ Following a ban, tobacco products are often stored within covered units, although typically remain at the point-of-sale and positioned below tobacco signage. This may maintain perceptions of tobacco availability and provide cues to tobacco use.^5^ However, there has been no similar ban on electronic cigarette (e-cigarette) retail displays, and a recent observational study in England found that highly visible, open displays of e-cigarette products were almost universal across tobacco retailers.^6^ Different regulatory approaches to tobacco and e-cigarette marketing may be appropriate, particularly to reflect the potential benefits of e-cigarette use as an aid to smoking cessation.^7^ However, further research is required to inform an approach that balances the potential impact on adult non-smokers and children of exposure to e-cigarette displays,^8^ which could undermine the benefits of a tobacco display ban.^4 9–11^

Children show high levels of recall for e-cigarette retail displays,^12^ which—in addition to recall of other e-cigarette media marketing—is associated with e-cigarette initiation.^13 14^ These associations are of similar magnitude to those found between tobacco retail display recall and smoking.^8^ Furthermore, exposure to e-cigarette marketing may impact *smoking*-related cognitions and behaviours (ie, a cross-cueing effect of exposure to one product on use of another).

There are several potential mechanisms through which exposure to e-cigarette retail displays could influence cognitions and, in turn, behaviour. One is through the normalisation of e-cigarette products, for example, via increasing product acceptability caused by higher visibility and marketing.^15^ This normalisation could influence initiation of, and susceptibility to, e-cigarettes products.^13 16^ Another is through cross-cueing effects, whereby the co-location of e-cigarette and tobacco products may lead to learned associations between these two categories of products, so that subsequent exposure to one product results in recall of the other.^17^ This recall could increase susceptibility to use through increased curiosity,^18^ as well as brand recognition and communication.^19^

An observational study of German children found that exposure to e-cigarette media advertisements was associated with use of e-cigarettes, tobacco cigarettes and hookah (waterpipe).^20^ Although these observational studies have the advantage of ecological validity, they also suffer from well-described problems regarding causal inference. Experimental studies can provide a stronger basis for causal inference, but can be challenging to conduct in real-world settings. Triangulating evidence from observational studies and experimental studies conducted in analogue (eg, online) settings can provide a more robust basis for informing policy and deciding whether there is a need to invest in real-world experimental studies (ie, field studies).

An experimental study in 2019 in an analogue setting—a replica convenience store—found that adolescents who were exposed to displays predominantly comprising tobacco products (where e-cigarettes comprised 10%–15% of the display area) were more willing to use e-cigarettes in the future, compared with those not exposed to the displays.^21^ However, there is currently a lack of experimental evidence, particularly in children, on the relationship between exposure to e-cigarette retail displays and susceptibility to, or attitudes towards, their use. This lack of evidence is unsurprising, given the practical difficulty of randomising children to different everyday marketing experiences. Nevertheless, it is possible to examine the impact of different exposures in the short-term in an analogue (eg, online) setting.

Experimental studies have not, as far as we are aware, focused on the impact of e-cigarette retail exposure in children on susceptibility and attitudes to tobacco smoking and e-cigarette use. Given that experimentation with cigarette smoking and vaping typically begins in adolescence,^22^ with two-thirds of UK adult smokers reporting that they took up smoking before the age of 18,^23^ we aimed to estimate the impact of analogue (ie, online) exposure to e-cigarette retail displays on susceptibility to smoking in children (aged 13–17 years) in the UK. Our secondary objectives were to estimate the impact of e-cigarette retail exposure on susceptibility to using e-cigarettes, and perceived harms of cigarettes and e-cigarettes. We examined the effects of both e-cigarette retail display visibility, and the proportion of retail displays made up of e-cigarettes products, based on evidence that altering the availability (both relative and absolute) of products such as food and alcohol and, by extension their visibility, is one potential intervention to change behaviours.^24 25^

## Methods

### Design

This was an online experimental study that used a 2 (e-cigarette retail display visibility: high vs low)×2 (proportion of e-cigarette images: 75% vs 25%) between-participants factorial design. Participants were randomised to one of four groups by an algorithm within the Qualtrics online platform (http://www.qualtrics.com/). The protocol was preregistered on the Open Science Framework (https://osf.io/pe34h/). The datasets generated and analysed during the current study are publicly available at the University of Bristol data repository.^26^

In each of the four groups, participants were shown 12 images of retail displays, which varied regarding the visibility of e-cigarette products and the proportion of e-cigarette images compared with unrelated products (eg, confectionery, stamps, drinks):

1. nine images of high visibility e-cigarette displays and three images of unrelated products;
2. three images of high visibility e-cigarette displays and nine images of unrelated products;
3. nine images of low visibility e-cigarette displays and three images of unrelated products;
4. three images of low visibility e-cigarette displays and nine images of unrelated products.

The study was designed to test three hypotheses:

H1: Children’s susceptibility to smoking is increased by exposure to higher *visibility* e-cigarette retail displays.

H2: Children’s susceptibility to smoking is increased by exposure to a higher *proportion* of e-cigarettes retail display images.

H3: There is an interaction between exposure to higher visibility e-cigarette retail displays and higher proportions of e-cigarettes display images, such that susceptibility to smoking is greatest when exposed to higher visibility displays with higher proportions of e-cigarette images, and lowest when exposed to lower visibility displays with lower proportions of e-cigarette images.

Further hypotheses were not prestated, although planned exploratory analyses were also conducted for susceptibility of e-cigarette use and perception of harm of smoking and e-cigarette use.

### Participants and recruitment

Participants were recruited by a research agency (MRFGR-UK: https://mrfgr.com/). All UK children aged 13–17 years were eligible to take part in the study. This age group of 13–17 years children was the youngest under-18 cohort available through the research agency. The research agency contacted their existing (adult) panel, which matches UK population in terms of age, gender, region and social economic status (n=20 000). Panel members were asked if they have children and, if so, their age. Those with children aged 13–17 years were provided with information about the study, as well as an invitation link, and invited to consent to their child taking part. If parental consent was given, parents were asked to pass on the study task for their children to complete.

One invitation link was sent per adult panel member; therefore, it was only possible for one child from each household to complete the task, and each child could only complete the task once. If a household had more than one eligible child, it was left to the discretion of the adult panel member to choose the child which took part. The children were given information about the study at the start of the online task and asked to provide assent to take part. Only children who provided assent continued to the study task. Debrief information was provided at the end of the task. The research agency received automated confirmation of study task completion, and credited panellists’ (ie, the parents or carers) accounts with points (which accumulate and can be redeemed for vouchers, etc). All data collected were anonymous; therefore, it was not possible for participants to withdraw after completing the study.

### Intervention

In each of the four study groups (see ‘Design’ section), participants were shown one of four image sets comprising 12 images of retail displays, using photographs taken in 2020 of the point-of-sale or aisle shelves from a range of UK supermarkets and convenience stores. Each image set included either high or low visibility e-cigarette retail displays, which made up a high (75%) or low (25%) proportion of the 12 images (see ‘Design’ section). E-cigarette proportion was based on the proportion of the 12 images that were of e-cigarettes, compared with unrelated products (eg, confectionery, stamps and drinks). A high e-cigarette proportion included nine images of e-cigarettes and three images of unrelated products, and a low proportion the opposite. E-cigarette display visibility was based on examples of retail e-cigarette displays with high (4–5) or low (1–2) conspicuousness scores, from a single checklist item from the DISPLAY visibility tool,^5^ as reported in a recent naturalistic observational study.^6^ For example, high visibility displays may be large, include many e-cigarette and related product options and colourful packages or signage, whereas low visibility displays may be small, located lower down behind the counter and offer few options.

We defined these ‘high’ and ‘low’ visibility categories based on the conspicuousness scores used in the naturalistic observational study,^6^ to enable comparability of findings across studies. The rationale for using ‘high’ and ‘low’ proportions of 25% and 75%, respectively was also for pragmatic reasons, to enable us to represent different degrees of short-term e-cigarette exposure.

### Measures

#### Demographic characteristics

All participants were asked their age (in years); sex (male, female, other, prefer not to say); ethnicity;^27^ and smoking and vaping history (online supplemental material).

10.1136/tobaccocontrol-2021-056980.supp1 Supplementary data

#### Primary outcome

The primary outcome of susceptibility to smoking was assessed among never smokers only, using an established validated measure.^28^ Participants were classified as either susceptible or not susceptible to smoking (online supplemental material).

#### Secondary outcomes

A susceptibility to using e-cigarettes measure was adapted from the susceptibility to smoking measure and assessed among never vapers only. A perception of harm of smoking was assessed among all participants using the following question:^29 30^ “How dangerous do you think it is to smoke one or two cigarettes occasionally?” (rated on a 5-point scale, 1=not very dangerous to 5=very dangerous), which was also adapted to assess perception of harm of e-cigarette use.

We prioritised susceptibility to smoking as our primary outcome and used perceptions of harm of smoking as a secondary outcome. Although both are predictors of smoking initiation,^31 32^ susceptibility is more proximal to the behaviour of concern, and in our view most relevant to the current policy debate.

#### Other measures

The frequency with which children visited supermarket and convenience stores was assessed using questions adapted from Edwards *et al* (online supplemental material).^4^ This study was conducted during the COVID-19 pandemic, which impacted typical routines for adults and children; therefore, we also assessed whether store visits had changed (online supplemental material).

### Procedure

Parents (or carers) who consented to their child taking part in the study were asked to pass the study over to their child to complete online, without others present, to encourage honest responses.

Participants were told that the study was about interest in products available in supermarkets and convenience stores, including snacks, drinks, cigarettes and e-cigarettes. Participants were informed that the study was completely anonymous, and no one, including their parents or carers, would be able to see their answers. Participants were asked to confirm their assent to continue via an online assent form and randomised to one of the four groups by the Qualtrics platform.

Participants were shown 12 images which appeared on the screen individually, in a random order, for at least 5-seconds before the participant was able to move on to the next image. After viewing all the images, participants were asked a series of questions, including free and cued recall of items presented, susceptibility to smoking and e-cigarette use, perceptions of harms of smoking and e-cigarette use, demographic information, attention checks, filler questions and history of smoking and vaping.^28^

At the end of the study, debrief information was provided for participants and their parents/carers, providing further details and the study rationale.

Data were collected in January 2021.

### Statistical analysis

#### Sample size determination

We adopted a pragmatic approach to sampling, to generate the most precise estimate of any effect of exposure to e-cigarette retail displays given the resources available. We were able to recruit approximately 1000 children (including all smoking and vaping experiences) via their parent’s membership of the research agency panel. We calculated that, for our primary outcome of susceptibility to smoking, a sample size of 187 per group would provide 80% power for a logistic regression to detect an effect size of OR 1.8 or larger (ie, from 38% to 52%, a change of 14%) using a two-sided test of a two-group between-subjects factor at an alpha level of 5%. Recruiting approximately 1000 participants would allow for up to a 25% attrition rate and lead to at least 750 with complete data for all outcomes (187/group)—see online supplemental material for further details.

#### Analysis

Logistic regression was used to calculate the odds of a child being susceptible to smoking or e-cigarette use, compared with being not susceptible. General linear models were used to assess differences in the perception of harm of smoking and e-cigarette use. Both analyses used a 2×2 design, with two independent binary factors of e-cigarette retail display visibility and proportion of e-cigarette images displayed, and a two-way interaction term. The interaction term was dropped in favour of a single model that included only the two main effects unless there was statistical evidence (defined as p<0.01) for an interaction. The two main effects of e-cigarette retail display visibility (reference category: low) and proportion of e-cigarette image (reference category: 25%) were calculated as either an OR or mean difference (MD), with associated 95% CI and p value.

Three subgroup analyses of the primary outcome of susceptibility to smoking were planned in order to exclude participants who: (i) reported visiting supermarkets or convenience stores more or less often than usual due to COVID-19; (ii) reported visiting supermarkets or convenience stores less than monthly and (iii) selected at least one ‘red herring’ item in the cued recall task (ie, the participant was deemed to not be paying attention to the task), as long as this exclusion did not reduce the sample by more than half (subgroup analysis (i) was not conducted for this reason). Model diagnostics were acceptable, although all logistic regression models predicted only one outcome. No formal subgroup testing adjustments were made.

All analyses were conducted in IBM SPSS V.27.

## Results

**Figure 1:**
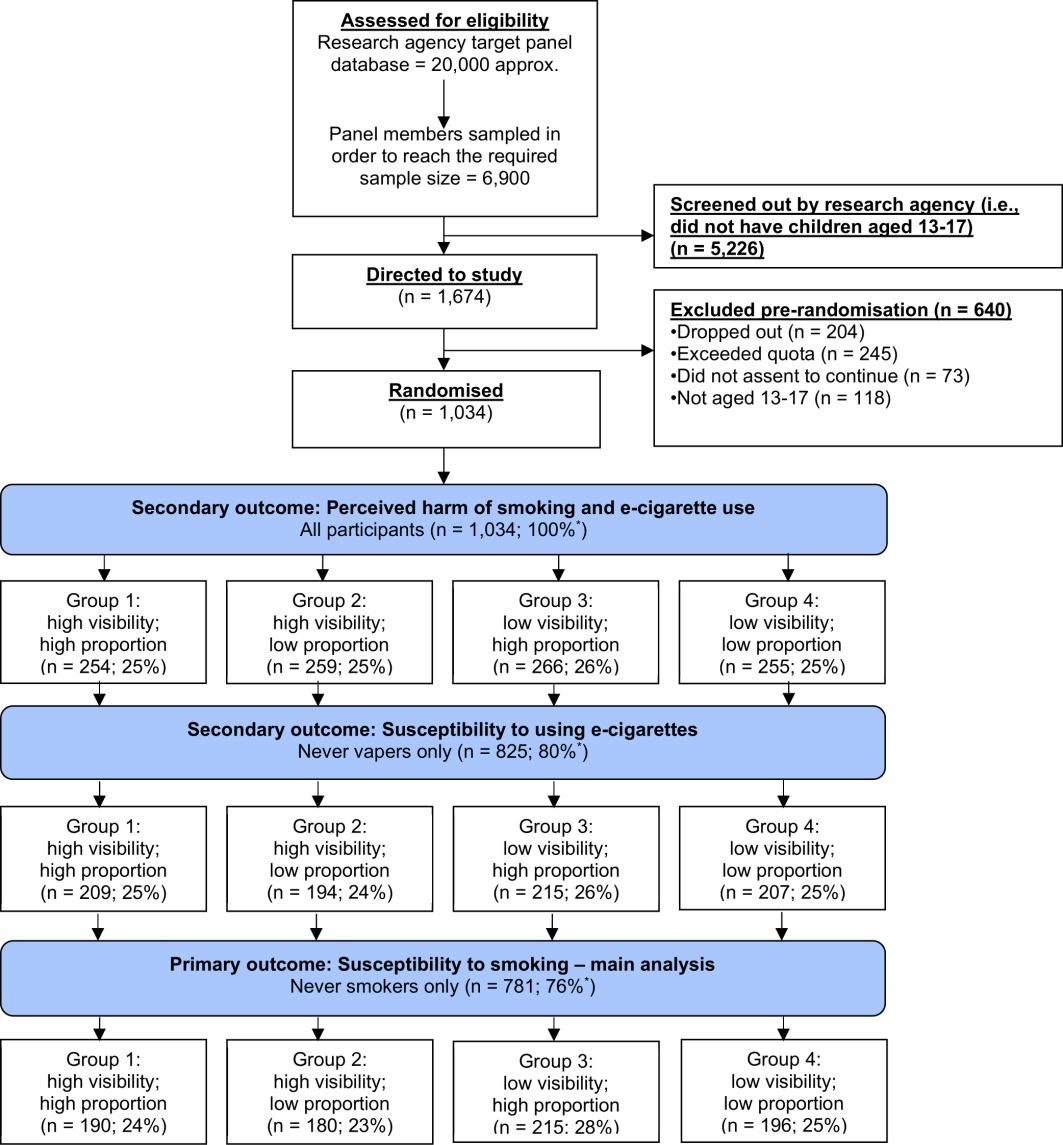
Consolidated Standards of Reporting Trials flow diagram. *Per cent of total sample.

The recruitment agency sampled approximately 6900 potential UK panel members, of whom 1674 were eligible (ie, had children aged 13–17 years), and were invited to consent to their child’s participation and redirected to the study (figure 1). Two hundred and four of the eligible panel members dropped out during the agency screening and did not start the study (figure 1). Of the participants who started the study, 436 were excluded as they either were not the required age (n=118), did not assent to take part (n=73), or were no longer required after the preset quota was reached (n=245) (figure 1).

In total, 1034 participants aged 13–17 years were randomised and completed the study. There were 781 participants (76%) included in the susceptibility to smoking main analysis (never smokers) and 825 participants (80%) included in the susceptibility to using e-cigarettes analysis (never vapers) (figure 1). All participants were included in the perceived harm of smoking and perceived harm of e-cigarette use analysis (figure 1).

The number of participants included in each analysis is shown in online supplemental table 1. Just under half of participants were female (45%), the majority reported that their ethnicity was white British (83%) and the mean age was 14.9 years (SD 1.3 years) (table 1).

**Table 1:** Demographic characteristics of participants in four groups varying in the visibility (high, low) and proportion of images (high, low) to which children were exposed

	High visibility; high proportion (n=254)	High visibility; low proportion (n=259)	Low visibility; high proportion (n=266)	Low visibility; low proportion (n=255)	Total (n=1034)
Gender—female (n, %)	114 (45%)	116 (45%)	123 (47%)	110 (43%)	463 (45%)
Ethnicity—White British (n, %)	209 (82%)	219 (85%)	221 (83%)	203 (80%)	852 (83%)
Age—years (M, SD)	14.8 (1.3)	15.0 (1.3)	14.8 (1.3)	15.0 (1.3)	14.9 (1.3)

M, mean; SD, standard deviation.

### Susceptibility to smoking and perceived harm of smoking

One-third of children (n=257, 33%) who reported never smoking were susceptible to smoking (table 2). Approximately one-quarter of children (n=237, 23%) reported low perception of harm of smoking (table 2). We did not find evidence to justify retaining the interaction term between e-cigarette visibility and the proportion of e-cigarette images displayed in either the susceptibility to smoking or perception of harm of smoking models (p=0.92 and p=0.097, respectively). Therefore, we dropped the interaction terms in favour of models estimating the two main effects only.

**Table 2:** Raw scores for the smoking and e-cigarette outcome measures for the four study groups varying in the visibility (high, low) and proportion of images (high, low) to which children were exposed

	High visibility; high proportion	High visibility; low proportion	Low visibility; high proportion	Low visibility; low proportion	Total
Susceptibility to smoking—yes* (n, %)	64 (34%)	50 (28%)	82 (38%)	61 (31%)	257 (33%)
Susceptibility to using e-cigarettes—yes† (n, %)	76 (36%)	61 (31%)	74 (34%)	63 (30%)	274 (33%)
Perceived harm of smoking—low‡ (n, %)	72 (29%)	69 (27%)	54 (21%)	42 (17%)	237 (23%)
Perceived harm of e-cigarette use—low‡ (n, %)	97 (39%)	92 (36%)	91 (35%)	78 (31%)	358 (35%)

*Scored ≥1 total to three questions relating to their smoking susceptibility, n=781 (never smokers only).

†Scored ≥1 total to three questions relating to their susceptibility to using e-cigarettes, n=825 (never vapers only).

‡Low perceived harm includes ‘not very dangerous’ (1) and ‘slightly dangerous’ (2) responses on a 5-point scale (1=not very dangerous to 5=very dangerous) of perceived harm n=1022 (all children).

Subgroup analyses were conducted for the primary outcome of susceptibility to smoking: (i) excluding children who reported visiting supermarkets or convenience stores less than once a month (remaining n=524/781 for analysis, 67%) and (ii) excluding those that were deemed to be not paying attention to the task (remaining n=676/781 for analysis, 87%).

#### Visibility

In the main analysis of the primary outcome (n=781), there was no evidence that e-cigarette retail display visibility influenced susceptibility to smoking (OR=0.84 (reference: low visibility, not susceptible), 95% CI 0.62 to 1.13, p=0.24). Similarly, there was no evidence of an effect on susceptibility to smoking in the subgroup analyses (visit frequency: OR=0.86 (reference: low visibility, not susceptible), 95% CI 0.56 to 1.32, p=0.49; attention check: OR=0.89 (reference: low visibility, not susceptible), 95% CI 0.65 to 1.23, p=0.49). However, there was evidence that greater visibility of e-cigarette retail displays reduced perceived harm of smoking (n=1034) (MD=−0.19 (reference: low visibility), 95% CI −0.34 to −0.04, p=0.016).

#### Proportion

In the main analysis of the primary outcome (n=781), there was evidence that the proportion of e-cigarette images displayed increased susceptibility to smoking (OR=1.34 (reference: low visibility, not susceptible), 95% CI 1.00 to 1.82, p=0.054). In our subgroup analyses, there was evidence that exposure to a higher proportion of e-cigarette images increased susceptibility to smoking among children who visited retail stores frequently (n=524, OR=1.59 (reference: low visibility, not susceptible), 95% CI 1.04 to 2.43, p=0.034), and among children who were paying attention (n=880, OR=1.43 (reference: low visibility, not susceptible), 95% CI 1.03 to 1.98, p=0.031). There was no evidence that the proportion of e-cigarette images displayed influenced perceived harm of smoking (n=1034) (MD=−0.07 (reference: low visibility), 95% CI −0.22 to 0.09, p=0.40).

### Susceptibility to using e-cigarettes and perceived harm of e-cigarette use

One-third of children (n=274, 33%) who reported never vaping were susceptible to e-cigarette use (table 2). Approximately one-third of children (n=358, 35%) reported low perception of harm of e-cigarette use (table 2). We did not find evidence to justify retaining the interaction term between e-cigarette visibility and the proportion of e-cigarette images displayed in either the susceptibility to e-cigarette use or perception of harm of e-cigarette use models (p=0.90 and p=0.19, respectively). Therefore, we dropped the interaction terms in favour of models estimating the two main effects only.

#### Visibility

There was no evidence for an effect on susceptibility to using e-cigarettes of e-cigarette retail display visibility (n=825) (OR=1.07 (reference: low visibility, not susceptible), 95% CI 0.80 to 1.43, p=0.65). There was also no evidence that perceived harm of e-cigarette use was influenced by e-cigarette retail display visibility (n=1034) (MD=−0.12 (reference: low visibility), 95% CI −0.28 to 0.05, p=0.16).

#### Proportion

There was no evidence for an effect on susceptibility to using e-cigarettes of proportion of e-cigarette images displayed (n=825) (OR=1.22 (reference: low visibility, not susceptible), 95% CI 0.91 to 1.64, p=0.18). There was also no evidence that perceived harm of e-cigarette use was influenced by the proportion of e-cigarette images displayed (n=1034) (MD=−0.10 (reference: low visibility), 95% CI −0.26 to 0.07, p=0.24).

All results are shown in online supplemental table 2.

## Discussion

In this online experimental study, we did not find evidence to suggest that e-cigarette retail display visibility influenced our primary outcome of children’s susceptibility to smoking, and only found weak evidence that the proportion of e-cigarette images displayed influenced smoking susceptibility. However, in subgroup analyses we found that children who visited retail stores more regularly, and who were deemed to be paying attention to the task, reported increased susceptibility to smoking after viewing a higher proportion of e-cigarette retail display images. There was no evidence of an interaction between exposure to high visibility e-cigarette retail displays and a higher proportion of e-cigarette images for smoking susceptibility in either the main or subgroup analysis. In exploratory analyses, for our secondary outcomes, we found evidence to suggest that high visibility e-cigarette displays reduce perceptions of smoking harms. Neither the visibility nor the proportion of e-cigarette retail display images shown appeared to influence susceptibility to using e-cigarettes or perceived harm of e-cigarette use.

As far as we are aware, this is the first experimental study to examine the potential impact of e-cigarette retail displays on children’s susceptibility to smoking. Nevertheless, the study has some limitations. First, we measured self-reported susceptibility and attitudes, rather than actual behaviour; however, these measures have been shown to predict subsequent behaviour.^28 33^ Extending this work to examine behaviour would be justified, although this would be challenging. Second, the effects we observed were only found in a subgroup analysis. These effects may be limited to children who visit retail stores regularly, but they should be treated with some caution and warrant replication. Third, we have previously reported that highly visible e-cigarette retail displays are near ubiquitous in two UK cities;^6^ therefore, the somewhat brief, single exposure used in this present study may have limited additional impact. Fourth, since e-cigarette products are often displayed alongside tobacco signage in real-world settings, and therefore in the images used in this study—and e-cigarettes are often positioned adjacent to tobacco storage units^6^—it may have been exposure to these smoking cues that increased smoking susceptibility. However, this also serves to highlight the potential consequences of co-locating products in this way, which future research should consider. Finally, information about the area in which participants lived, including information on socioeconomic position, was a non-mandatory field in the research agency’s demographic screening, and therefore was only available for a subset of participants. We were therefore unable to explore any modifying effect of socioeconomic position, due to the low statistical power available and the potential selection bias among those who had chosen to provide information. The impact of deprivation should be considered in future research given that several studies report increased visibility, recall and use of tobacco products and e-cigarettes in deprived areas.^12 34–37^

Despite these limitations, this study extends evidence from observational studies of the potential impact of children’s exposure to product marketing via retail displays.^8 12–14^ These studies all generally explored cueing effects of exposure to one product on cognitions for that product, rather than cross-cueing effects (eg, of e-cigarette retail exposure on smoking cognitions). Notably, we did not find evidence of a direct cueing effect of e-cigarette exposure on product-specific cognitions (ie, exposure to e-cigarette cues did not appear to influence susceptibility to using e-cigarettes or perceptions of harm). These differences may be due to the different study designs. For example, the association between e-cigarette exposure and e-cigarette cognitions and behaviours found in observational studies may not be causal. It is also possible that the single brief exposure used in our online study may be insufficient to elicit an effect. Nevertheless, we did find evidence of cross-cueing effects, which suggests that single and/or brief exposure may have some impact. This is consistent with observational studies examining exposure to e-cigarette advertisements.^20^ The cross-cueing effects we observed are also consistent with other experimental studies examining the impact of exposure to e-cigarette advertisements.^29^ This study therefore extends the existing literature on cross-cueing effects, providing reasons to be concerned about the potential impact of e-cigarette marketing in retail settings.

The results of our subgroup analysis of the primary outcome, and the analysis of the secondary outcome of perceived harm of smoking, demonstrate a pattern of concern, given that both susceptibility to smoking^31^ and perceived harm of smoking^32^ are predictors of smoking initiation among children. The fact that our subgroup analyses were planned raises confidence in these findings but warrant replication to raise confidence in their robustness. As we note above, in this study, children were exposed to e-cigarette images only briefly, and during a single session. However, a 2019 study in Scotland indicated that children aged 10–11 years are exposed to tobacco retailing environments for an average of 23 minutes per week, across an average of 43 independent encounters.^36^ Moreover, children from the most deprived areas accumulated six times this duration (and seven times this frequency) of exposure, compared with children from the least deprived areas.^36^ The potential impact of everyday exposure to e-cigarette displays on attitudes towards smoking should therefore be a key consideration in regulatory decisions regarding e-cigarette marketing, in order to balance the potential benefits of e-cigarettes as smoking cessation aids for adults against potential effects on smoking attitudes and behaviour among children.^7^

## Conclusion

This study provides some evidence that exposing children to e-cigarette retail displays may increase their susceptibility to smoking and reduce their perceptions of the harms of smoking. The fact that we did not find evidence to suggest an effect of e-cigarette retail display exposure on susceptibility to using e-cigarettes is encouraging, given concerns about e-cigarette use in children. However, the evidence we find for cross-cueing effects provides grounds for remaining cautious about the impact of the introduction of new products to market, and associated marketing strategies.^38^ If e-cigarette displays have an impact on smoking attitudes—and potentially behaviour—in children, this is obviously a matter of public health concern. A review of the current regulatory discrepancy between tobacco and e-cigarette point-of-sale marketing is warranted.

**Key messages**

**What is already known on this topic**

- Many countries, including the UK, have banned tobacco retail displays because of links to increased tobacco purchasing and reduced smoking cessation; however, there has been no such ban on e-cigarette retail displays in the UK.
- Current evidence suggests an association between exposure to products in retail settings and susceptibility to their use, as well as evidence that exposure to one product (eg, e-cigarettes) may influence use of another related product (eg, tobacco cigarettes).
- There is an absence of evidence on the effects of exposure to e-cigarette retail displays in children, which this study aims to address.

**What this study adds**

- Exposure to e-cigarette retail displays may increase susceptibility to smoking in a subset of children, while exposure to higher visibility e-cigarette retail displays may reduce perceived harms of smoking.

**How this study might affect research, practice and/or policy**

- A review of the current regulatory discrepancy between tobacco and e-cigarette point-of-sale marketing is warranted, given their potential to encourage smoking in children.

## Data Availability

Data are available in a public, open access repository. The datasets generated and analysed during the current study are available at the University of Bristol data repository, data.bris, at: 10.5523/bris.glkgioxkg9ue2gidk6gktm7pd
