# Supplementary material for: Impact of e-cigarette retail displays on attitudes to smoking and vaping in children: an online experimental study
Source: Tob Control. 2022 Apr 13;32(e2):e220–7. doi: 10.1136/tobaccocontrol-2021-056980 (PMC10423515; doi:10.1136/tobaccocontrol-2021-056980)
Supplement: Supplementary data [file tobaccocontrol-2021-056980supp001.pdf]

Supplementary Material

Supplementary Methods

Ethnicity

Self-assigned ethnicity was coded as follows: White: 1. English / Welsh / Scottish / Northern Irish / British, 2. Irish, 3. Gypsy or Irish Traveller, 4. Any other White background, please describe; Mixed / Multiple ethnic groups: 5. White and Black Caribbean, 6. White and Black African, 7. White and Asian, 8. Any other Mixed / Multiple ethnic background, please describe; Asian / Asian British: 9. Indian, 10. Pakistani, 11. Bangladeshi, 12. Chinese, 13. Any other Asian background, please describe; Black / African / Caribbean / Black British: 14. African, 15. Caribbean, 16. Any other Black / African / Caribbean background, please describe; Other ethnic group: 17. Arab; 18. Any other ethnic group, please describe.

Primary outcome

The primary outcome of susceptibility to smoking was assessed among never smokers only, using an established validated measure with three questions:[1] 1) "Do you think that you will try a cigarette soon?" (yes / no); 2) "If one of your best friends were to offer you a cigarette, would you smoke it?" (definitely not / probably not /probably yes / definitely yes); and 3) "Do you think you will be smoking cigarettes one year from now?" (definitely not / probably not / probably yes / definitely yes). Participants were classified as not susceptible to smoking. if they answered 'no' to question one and 'definitely not' to questions two and three. Participants were classified as susceptible to smoking if they gave any other answers.

This measure of susceptibility to smoking,[1] has been shown to reliably predict future smoking behaviour: susceptible never smokers are twice as likely to become established smokers compared to non-susceptible never smokers (OR 2.1, 95% CI 1.6, 2.7).[2] This measure is commonly used in studies investigating the impact of tobacco and e-cigarette marketing on children and young people.[3-8] In a study by Vasiljevic and colleagues, 38% of children in the control group were susceptible to smoking.[5]

Procedure

To reduce participants' attention to smoking and vaping items, outcome measures were presented embedded within broader filler questions about other products available in supermarkets and convenience stores (e.g., sweet and savoury snacks, fizzy and energy drinks). To encourage engagement with the images, participants were told that their memory for items would be tested. After viewing the image sets, they were asked to record all items they could remember without prompting (free recall), and then record items they could remember from a list of possible items (cued recall). The cued recall task included a number of 'red herrings' to check whether participants were paying attention to the task (i.e., if a participant incorrectly selected an item which was not shown to them previously). Finally, participants were given an option to record their views on the impact of retail displays on their interest in trying and buying products in free-text comments.

Blackwell AKM, *et al. Tob Control* 2023; 32:e220–e227. doi: 10.1136/tobaccocontrol-2021-056980

Other measures

Smoking history was assessed by asking: 1) "Have you ever smoked a cigarette?", and 2) "Have you ever tried or experimented with cigarette smoking, even a few puffs?". Those who answered ‘no’ to both questions were classified as never smokers. These questions were also adapted for assessing vaping history by asking: 1) “Have you ever used an e-cigarette (vape)?”, and 2) “Have you ever tried or experimented with an e-cigarette, even a few puffs?”. Those that answered ‘no’ to both questions were classified as never vapers.

The frequency with which children visited supermarket and convenience stores was assessed using questions adapted from Edwards and colleagues.[9] “How often do you visit: a. the supermarket (e.g., Tesco, Asda or Sainsbury’s); b. a convenience store (e.g., Spar, McColl’s, Costcutter, or other local corner shop/newsagent)?” (never / less than once a month / a few times a month / once a week / a few times a week / most days of the week / every day of the week).

This study was conducted during the Covid-19 pandemic, which impacted typical routines for adults and children; therefore, we also assessed whether store visits had changed: “Has this changed because of Covid-19?" (Yes, I visit [supermarkets/convenience stores] less than I did before Covid-19; Yes, I visit [supermarkets/convenience stores] more than I did before Covid-19; No, I visit [supermarkets/convenience stores] the same number of times as I did before Covid-19).

Public and patient involvement

Prior to the study commencing, we sought and obtained feedback about the experimental task from three primary and secondary school teachers, and three children (aged 13-17 years), all of whom were personal contacts of the research team. Adjustments to the task were made based on this feedback, such as to the wording of instructions and the layout. Feedback was also given about the length of time taken to complete the task, level of engagement required, and degree of participant burden. Pre-defined outcome measures were discussed with children and teachers, but this did not result in any change to the measures used. Unfortunately, because participants were recruited via a research agency, it was not possible to include them in any dissemination.

Blackwell AKM, *et al. Tob Control* 2023; 32:e220–e227. doi: 10.1136/tobaccocontrol-2021-056980

Supplementary Table S1. Number of participants analysed between study groups for all analyses.

	Number of participants in each study group that were analysed (%)[1]				Total
**Analysis**	**Group 1: high visibility; high proportion**	**Group 2: high visibility; low proportion**	**Group 3: low visibility; high proportion**	**Group 4: low visibility; low proportion**	**(% of total sample [n=1034] unless otherwise stated)**
**Susceptibility to smoking** – main analysis (never smokers only)	190 / 781 (24%)	180 / 781 (23%)	215 / 781 (28%)	196 / 781 (25%)	781 /1034 (76%)
**Susceptibility to smoking** – subgroup analysis (never smokers only)[2]	121 / 524 (48%)	129 / 524 (51%)	140 / 524 (53%)	134 / 524 (53%)	524 / 781 (67%)
**Susceptibility to smoking** – subgroup analysis (never smokers only)[3]	175 / 676 (26%)	144 / 676 (21%)	194 / 676 (29%)	163 / 676 (24%)	676 / 781 (87%)
**Perceived harm of smoking** – main analysis (all participants)	254 / 1034 (25%)	259 / 1034 (25%)	266 / 1034 (26%)	255 / 1034 (25%)	1034 / 1034 (100%)
**Susceptibility to using e-cigarettes** – main analysis (never vapers only)	209 / 825 (25%)	194 / 825 (24%)	215 / 825 (26%)	207 / 825 (25%)	825 / 1034 (80%)
**Perceived harm of e-cigarette use** – main analysis (all participants)	254 / 1034 (25%)	259 / 1034 (25%)	266 / 1034 (26%)	255 / 1034 (25%)	1034 / 1034 (100%)

[1] the number of participants that were analysed in each study group (denominator is the total number analysed)
[2] excluding children who reported visiting supermarkets or convenience stores less than once a month (total % is out of 781)
[3] excluding those that were deemed to be not paying attention to the task (total % is out of 781)

Blackwell AKM, *et al. Tob Control* 2023; 32:e220–e227. doi: 10.1136/tobaccocontrol-2021-056980

Supplementary Table S2. Results of all analyses.

	**Visibility**			**Proportion**		
	**High n (%)**	**Low* n (%)**	**OR^ (95% CI) P value**	**High n (%)**	**Low* n (%)**	**OR^ (95% CI) P value**
**Susceptibility to smoking (yes)**[1] – main analysis (never smokers only)	114 (31%)	143 (35%)	0.84 (0.62, 1.13) p=0.24	146 (36%)	111 (30%)	1.34 (1.00, 1.82) p=0.054
**Susceptibility to smoking (yes)**[1] – subgroup analysis (never smokers only)[2]	61 (37%)	81 (41%)	0.86 (0.56, 1.32) p=0.49	83 (45%)	59 (36%)	1.59 (1.04, 2.43) p=0.034
**Susceptibility to smoking (yes)**[1] – subgroup analysis (never smokers only)[3]	102 (32%)	123 (35%)	0.89 (0.65, 1.23) p=0.49	136 (37%)	89 (29%)	1.43 (1.03, 1.98) p=0.031
**Susceptibility to using e-cigarettes (yes)**[4] – main analysis (never vapers only)	137 (34%)	137 (33%)	1.07 (0.80, 1.43) p=0.65	150 (35%)	124 (31%)	1.22 (0.91, 1.64) p=0.18
**Perceived harm of smoking (low)**[5] – main analysis (all participants)	141 (28%)	96 (18%)	-0.19^ (-0.34, -0.04) p=0.016	126 (24%)	111 (22%)	-0.07^ (-0.22, 0.09) p=0.40
**Perceived harm of e-cigarette use (low)**[5] – main analysis (all participants)	189 (37%)	169 (32%)	-0.12^ (-0.28, 0.05) p=0.16	188 (36%)	170 (33%)	-0.10^ (-0.26, 0.07) p=0.24

*Reference category is always low visibility, and where logistic regression was used, the reference category was 'not susceptible'.
Odds ratio (OR). Confidence interval (CI).
^Unless otherwise stated, where value is a MD (mean difference).
[1] scored ≥ 1 total to three questions relating to their smoking susceptibility
[2] excluding children who reported visiting supermarkets or convenience stores less than once a month (total % is out of 781)
[3] excluding those that were deemed to be not paying attention to the task (total % is out of 781)
[4] scored ≥ 1 total to three questions relating to their vaping susceptibility
[5] low perceived harm: includes 'not very dangerous' (1) and 'slightly dangerous' (2) responses on 5-point scale (1=not very dangerous to 5=very dangerous) of perceived harm

Blackwell AKM, *et al. Tob Control* 2023; 32:e220–e227. doi: 10.1136/tobaccocontrol-2021-056980

References

1. Pierce JP, Choi WS, Gilpin EA, et al. Validation of susceptibility as a predictor of which adolescents take up smoking in the United States. *Psychol Health* 1996;15(5):355-61. doi: 10.1037/0278-6133.15.5.355
2. Choi WS, Gilpin EA, Farkas AJ, et al. Determining the probability of future smoking among adolescents. *Addiction* 2001;96(2):313-23. doi: 10.1046/j.1360-0443.2001.96231315
3. Mackintosh AM, Moodie C, Hastings G. The association between point-of-sale displays and youth smoking susceptibility. *Nicotine Tob Res* 2012;14(5):616-20. doi: 10.1093/ntr/ntr185
4. Pasch KE, Nicksic NE, Opara SC, et al. Recall of Point-of-Sale Marketing Predicts Cigar and E-Cigarette Use Among Texas Youth. *Nicotine Tob Res* 2018;20(8):962-69. doi: 10.1093/ntr/ntx237
5. Vasiljevic M, St John Wallis A, Codling S, et al. E-cigarette adverts and children's perceptions of tobacco smoking harms: an experimental study and meta-analysis. *BMJ Open* 2018;8(7):e020247. doi: 10.1136/bmjopen-2017-020247
6. Paynter J, Edwards R, Schluter PJ, et al. Point of sale tobacco displays and smoking among 14–15 year olds in New Zealand: a cross-sectional study. *Tob Control* 2009;18(4):268-74. doi: 10.1136/tc.2008.027482
7. Spanopoulos D, Britton J, McNeill A, et al. Tobacco display and brand communication at the point of sale: implications for adolescent smoking behaviour. *Tob Control* 2014;23(1):64-9. doi: 10.1136/tobaccocontrol-2012-050765
8. Martino SC, Setodji CM, Dunbar MS, et al. Increased attention to the tobacco power wall predicts increased smoking risk among adolescents. *Addict Behav* 2019;88:1-5. doi: 10.1016/j.addbeh.2018.07.024
9. Edwards R, Ajmal A, Healey B, et al. Impact of removing point-of-sale tobacco displays: data from a New Zealand youth survey. *Tob Control* 2017;26(4):392-98. doi: 10.1136/tobaccocontrol-2015-052764
